# Supplementary material for: Quantification of cerebrospinal fluid tumor DNA in lung cancer patients with suspected leptomeningeal carcinomatosis
Source: NPJ Precis Oncol. 2024 May 28;8:121. doi: 10.1038/s41698-024-00582-1 (PMC11133465; doi:10.1038/s41698-024-00582-1)
Supplement: Supplementary file 1 — Supplement [file 41698_2024_582_MOESM1_ESM.pdf]

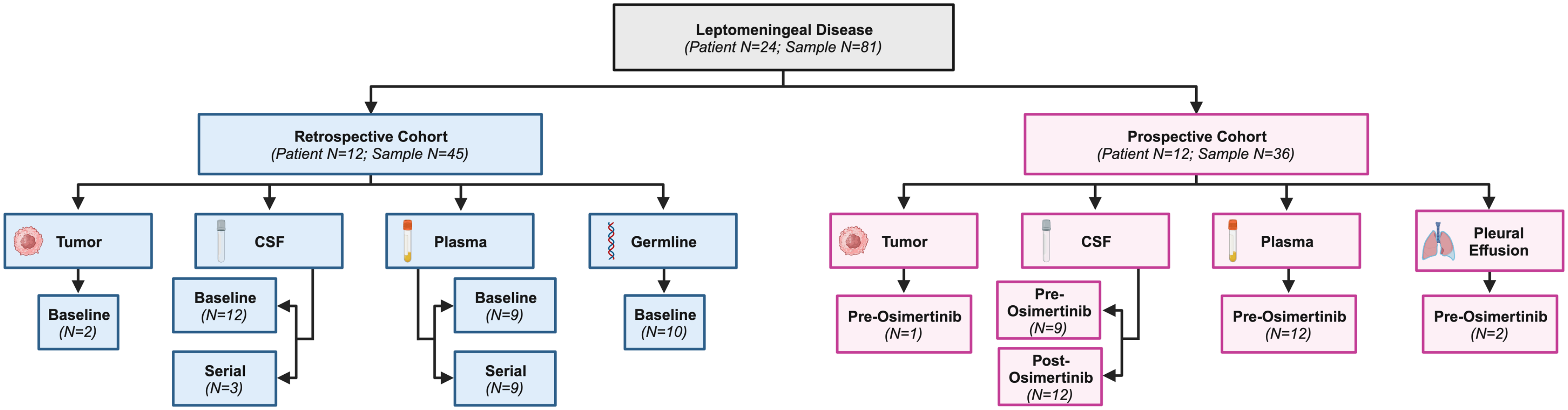

Supplementary Figure 1. Schematic overview of study patients and samples.

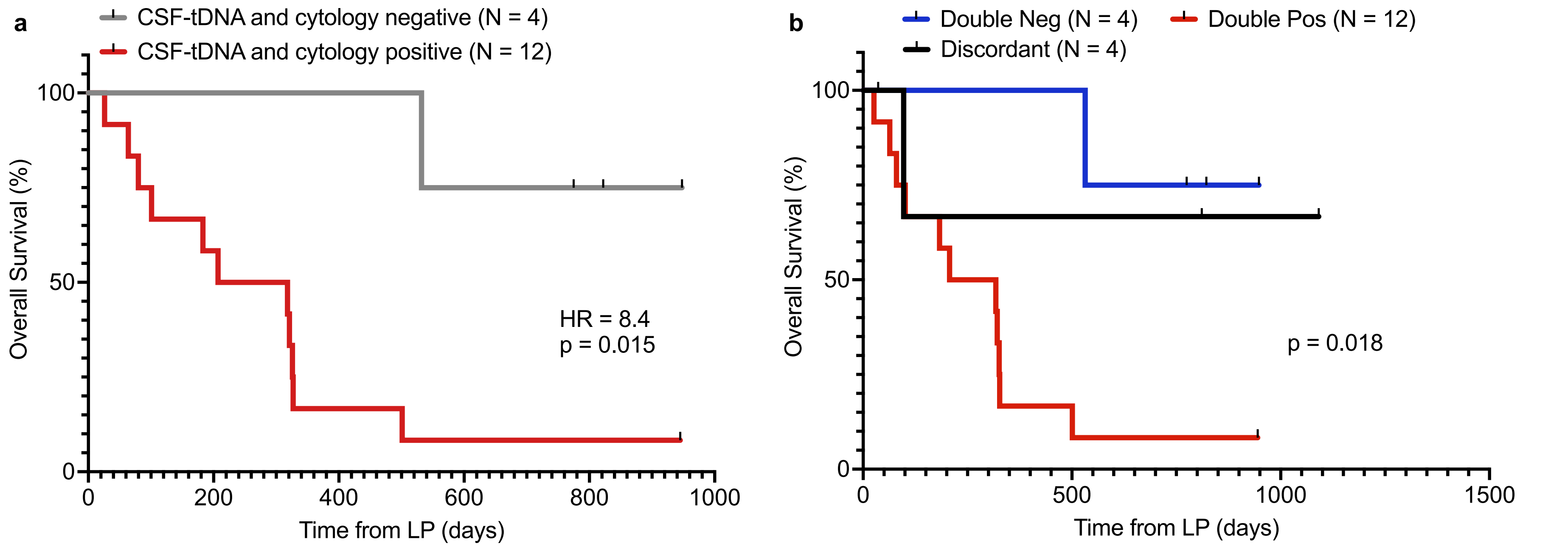

Supplementary Figure 2. (A) Kaplan-Meier curve comparing patients with detectable CSF-tDNA and cytology positive (N = 12) and undetectable CSF-tDNA and cytology negative (N = 4) at first lumbar puncture for the endpoint of overall survival (P = 0.015, HR = 8.5). P value and hazard ratio were calculated from the log-rank test. (B) Kaplan-Meier curve comparing patients with CSF-tDNA and cytology negative (Double neg, N=4), patients with CSF-tDNA and cytology positive (Double pos, N=12), and patients with CSF-tDNA positive and cytology negative (Discordant, N=4) for the endpoint of overall survival (P = 0.018). P value calculated from the log-rank test.

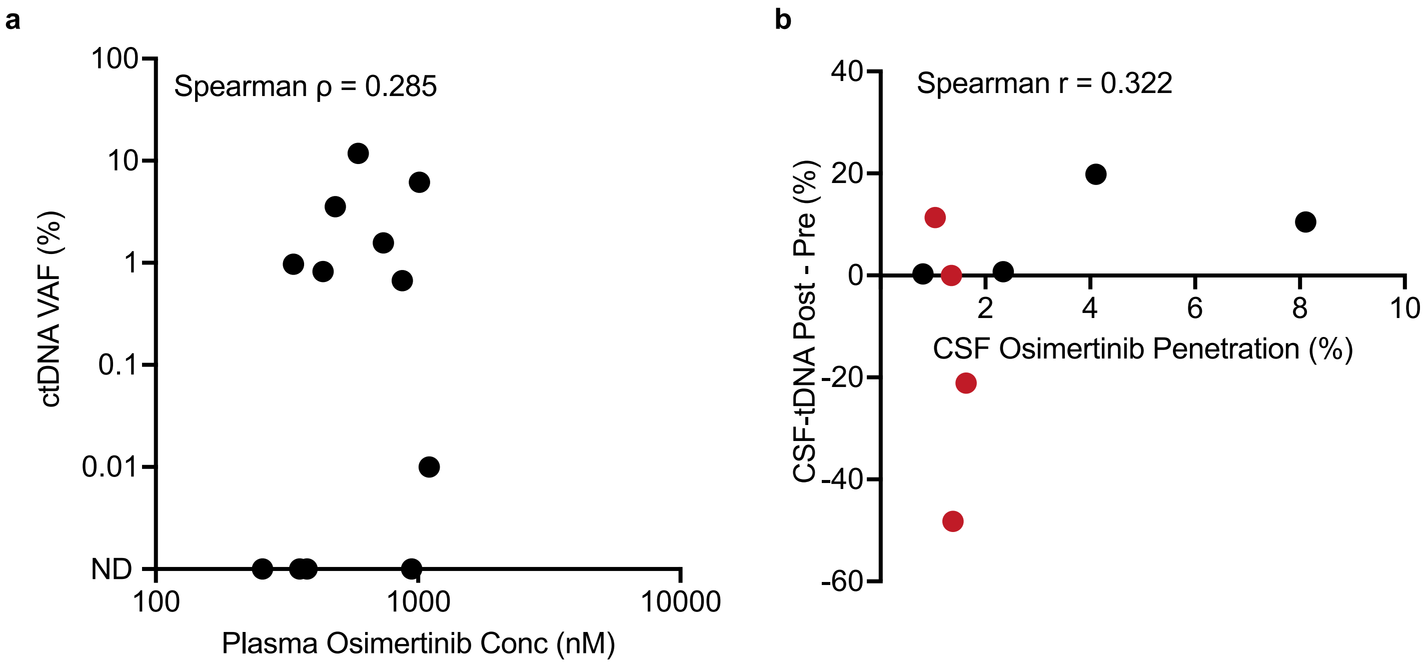

Supplementary Figure 3. (A) Correlation of post-osimertinib ctDNA VAF %) with plasma osimertinib concentration (nM). ρ was calculated by Spearman correlation. (B) Correlation between osimertinib CSF penetration and the difference in on-osimertinib and pre-osimertinib VAF. ρ was calculated by Spearman correlation

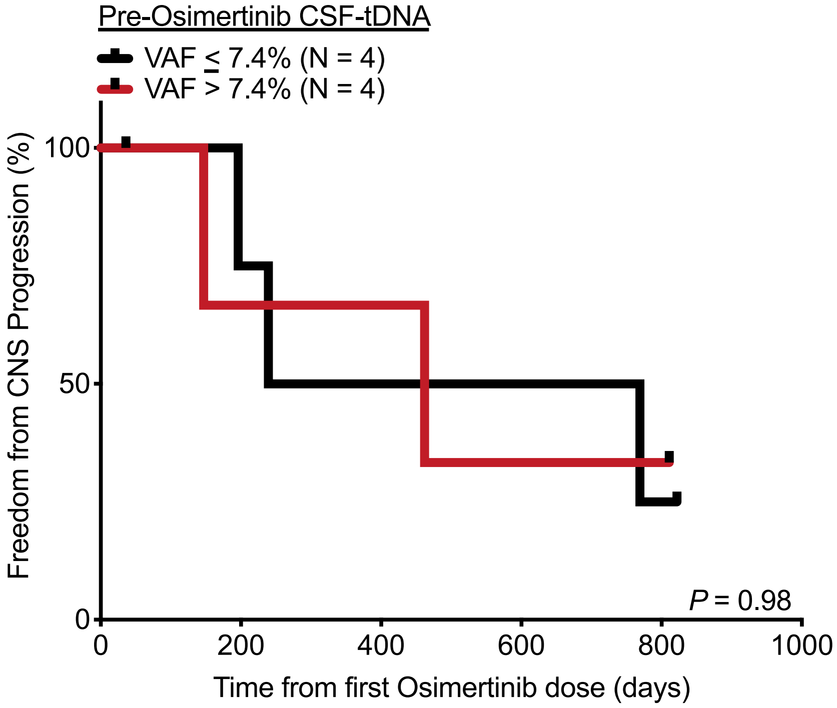

Supplementary Figure 4. Kaplan-Meier curve comparing patients with pre-osimertinib CSF-tDNA VAF above and below median (7.4%) for endpoint of freedom from CNS progression (P =0.98). P value calculated from the log-rank test.
