# Supplementary material for: Quantification of cerebrospinal fluid tumor DNA in lung cancer patients with suspected leptomeningeal carcinomatosis
Source: NPJ Precis Oncol. 2024 May 28;8:121. doi: 10.1038/s41698-024-00582-1 (PMC11133465; doi:10.1038/s41698-024-00582-1)
Supplement: Supplementary file 3 — Reporting Summary [file 41698_2024_582_MOESM3_ESM.pdf]

nature portfolio

Corresponding author(s): Tej D. Azad

Last updated by author(s): 12/29/2024

Reporting Summary

Nature Portfolio wishes to improve the reproducibility of the work that we publish. This form provides structure for consistency and transparency in reporting. For further information on Nature Portfolio policies, see our Editorial Policies and the Editorial Policy Checklist.

Statistics

For all statistical analyses, confirm that the following items are present in the figure legend, table legend, main text, or Methods section.

n/a	Confirmed	
☐	☒	The exact sample size (*n*) for each experimental group/condition, given as a discrete number and unit of measurement
☐	☒	A statement on whether measurements were taken from distinct samples or whether the same sample was measured repeatedly
☐	☒	The statistical test(s) used AND whether they are one- or two-sided<br>*Only common tests should be described solely by name; describe more complex techniques in the Methods section.*
☐	☒	A description of all covariates tested
☐	☒	A description of any assumptions or corrections, such as tests of normality and adjustment for multiple comparisons
☐	☒	A full description of the statistical parameters including central tendency (e.g. means) or other basic estimates (e.g. regression coefficient) AND variation (e.g. standard deviation) or associated estimates of uncertainty (e.g. confidence intervals)
☐	☒	For null hypothesis testing, the test statistic (e.g. *F*, *t*, *r*) with confidence intervals, effect sizes, degrees of freedom and *P* value noted<br>*Give P values as exact values whenever suitable.*
☐	☒	For Bayesian analysis, information on the choice of priors and Markov chain Monte Carlo settings
☐	☒	For hierarchical and complex designs, identification of the appropriate level for tests and full reporting of outcomes
☐	☒	Estimates of effect sizes (e.g. Cohen's *d*, Pearson's *r*), indicating how they were calculated

Our web collection on statistics for biologists contains articles on many of the points above.

Software and code

Policy information about availability of computer code

Data collection	No software used for data collection.
Data analysis	All statistical analyses were done using Prism 7 (GraphPad Software) or R v3.2.2 (http://www.r-project.org) through the RStudio environment. Visualizations created with BioRender.com.

For manuscripts utilizing custom algorithms or software that are central to the research but not yet described in published literature, software must be made available to editors and reviewers. We strongly encourage code deposition in a community repository (e.g. GitHub). See the Nature Portfolio guidelines for submitting code & software for further information.

Data

Policy information about availability of data

All manuscripts must include a data availability statement. This statement should provide the following information, where applicable:

- Accession codes, unique identifiers, or web links for publicly available datasets
- A description of any restrictions on data availability
- For clinical datasets or third party data, please ensure that the statement adheres to our policy

Reasonable requests for additional code and data access will be reviewed by the senior authors to determine whether they can be fulfilled in accordance with privacy restrictions. Requests for additional materials related to this work should be directed to M.D. and T.D.A.

Research involving human participants, their data, or biological material

Policy information about studies with human participants or human data. See also policy information about sex, gender (identity/presentation), and sexual orientation and race, ethnicity and racism.

Reporting on sex and gender	Acknowledged
Reporting on race, ethnicity, or other socially relevant groupings	Acknowledged
Population characteristics	Described in our manuscript.
Recruitment	The samples analyzed in this article were collected at two institutions. Thirteen patients were recruited at Stanford Hospital and Clinics and underwent lumbar puncture as part of routine clinical management of suspected LMD. Samples from twelve patients who were enrolled in a prospective study to determine the efficacy of osimertinib in the treatment of refractory LMD at the Institute of Biomedical Research and Innovation Hospital were also included.44 In the prospective cohort, collected and analyzed samples included: a pre-osimertinib sample (CSF, N = 9; tumor, N = 1; pleural effusion, N = 2), a post-osimertinib CSF sample (N = 12), and a time-matched post-osimertinib plasma sample (N = 12). The post-osimertinib samples were collected either at time of disease progression or at last available follow up. This prospective cohort is referred to as the "prospective osimertinib cohort" throughout the manuscript. In both cohorts, MRI was obtained prior to lumbar puncture. Informed consent was obtained from all patients and enrollment of each cohort was approved by the institutional review board at each respective institution. Detailed information regarding patient and samples is depicted in Supplementary Figure 1.
Ethics oversight	Informed consent was obtained from all patients and enrollment of each cohort was approved by the institutional review board at each respective institution

Note that full information on the approval of the study protocol must also be provided in the manuscript.

Field-specific reporting

Please select the one below that is the best fit for your research. If you are not sure, read the appropriate sections before making your selection.

☒ Life sciences ☐ Behavioural & social sciences ☐ Ecological, evolutionary & environmental sciences

For a reference copy of the document with all sections, see nature.com/documents/nr-reporting-summary-flat.pdf

Life sciences study design

All studies must disclose on these points even when the disclosure is negative.

Sample size	Included in our manuscript
Data exclusions	Included in our manuscript
Replication	Included in our manuscript
Randomization	Not relevant as our study was not a clinical trial and enrolled a non-randomized prospective cohort for study.
Blinding	Not relevant as our study was not a clinical trial and enrolled a non-blinded prospective cohort for study.

Reporting for specific materials, systems and methods

We require information from authors about some types of materials, experimental systems and methods used in many studies. Here, indicate whether each material, system or method listed is relevant to your study. If you are not sure if a list item applies to your research, read the appropriate section before selecting a response.

Materials & experimental systems

n/a	Involved in the study
☒	☐ Antibodies
☒	☐ Eukaryotic cell lines
☒	☐ Palaeontology and archaeology
☒	☐ Animals and other organisms
☐	☒ Clinical data
☒	☐ Dual use research of concern
☒	☐ Plants

Methods

n/a	Involved in the study
☒	☐ ChIP-seq
☒	☐ Flow cytometry
☒	☐ MRI-based neuroimaging

Clinical data

Policy information about clinical studies

All manuscripts should comply with the ICMJE guidelines for publication of clinical research and a completed CONSORT checklist must be included with all submissions.

Clinical trial registration: N/A

Study protocol: This was not a clinical trial

Data collection: The samples analyzed in this article were collected at two institutions between 2015 and 2020. . Thirteen patients were recruited at Stanford Hospital and Clinics and underwent lumbar puncture as part of routine clinical management of suspected LMD. Samples from twelve patients who were enrolled in a prospective study to determine the efficacy of osimertinib in the treatment of refractory LMD at the Institute of Biomedical Research and Innovation Hospital were also included.44 In the prospective cohort, collected and analyzed samples included: a pre-osimertinib sample (CSF, N = 9; tumor, N = 1; pleural effusion, N = 2), a post-osimertinib CSF sample (N = 12), and a time-matched post-osimertinib plasma sample (N = 12). The post-osimertinib samples were collected either at time of disease progression or at last available follow up. This prospective cohort is referred to as the “prospective osimertinib cohort” throughout the manuscript. In both cohorts, MRI was obtained prior to lumbar puncture.

Outcomes: Definition of definitive (1) and possible (2A and 2B) leptomeningeal disease
LMD diagnosis was considered definitive under the following conditions:
1. Positive CSF cytology or positive clinical EGFR CSF PCR in the initial LP OR
2A. MRI of the brain or spine performed prior to the diagnostic LP with unequivocal evidence of LMD AND
2B. Progressive neurological symptoms consistent with LMD, following exclusion of other possible causes
MRIs were reviewed for evidence of LMD by a board-certified neuroradiologist (M.I). This definition was adapted based on a prior study evaluating a flow cytometry-based method for LMD diagnosis.61

Plants

Seed stocks: N/A

Novel plant genotypes: N/A

Authentication: N/A
